# Clinical and immunomicrobiome correlates of a standardized Qingpao Chushi Jiedu Fang regimen in palmoplantar pustulosis

**DOI:** 10.3389/fmed.2026.1852035

**Published:** 2026-07-03

**Authors:** Junhui Wang, Yannan Yang, Zehui Chen

**Affiliations:** 1 Guang’AnMen Hospital, China Academy of Chinese Medical Sciences, Beijing, China; 2 Institute of Information on Traditional Chinese Medicine, China Academy of Chinese Medical Sciences, Beijing, China

**Keywords:** 16S rDNA, clinical trial, dampness-resolving and detoxifying method, oral microbiome, palmoplantar pustulosis, traditional Chinese medicine

## Abstract

**Background:**

Palmoplantar pustulosis (PPP) is a chronic inflammatory dermatosis with limited therapeutic options. Traditional Chinese Medicine (TCM) formulations may benefit PPP, yet microbiome-immune mechanisms underlying clinical response remain unclear.

**Objectives:**

To evaluate whether an 8-week standardized Qingpao Chushi Jiedu Fang (QCF) regimen is associated with coordinated changes in clinical severity, oral microbiota, and circulating cytokines in PPP.

**Methods:**

Thirty PPP patients received an 8-week standardized Qingpao Chushi Jiedu Fang (QCF) regimen. Clinical severity (PPPASI, Palmoplantar Pustulosis Area and Severity Index; Dermatology Life Quality Index, DLQI; pruritus/pain Visual Analogue Scale, VAS), oral microbiota (16S rDNA sequencing), and serum cytokines (IL-1β, IL-4, IFN-*α*, IFN-*γ*) were assessed before and after treatment. Subgroup analyses were performed by smoking status.

**Results:**

PPPASI significantly decreased from week 2 onward (*p* < 0.001), with further improvement at weeks 4 and 8, while week-6 vs. week-4 changes were non significant (*p* > 0.05). DLQI declined significantly at weeks 4–8 (*p* < 0.05). Pain scores showed improvement only at week 6 vs. week 2 (*p* < 0.05), and itch scores improved at week 8 (*p* < 0.05). Oral microbial *α*- and *β*-diversity shifted significantly after treatment (*p* < 0.05), with clear changes in community structure and taxa; smokers exhibited more pronounced restructuring. Cytokine levels changed concordantly, with IL-1β, IL-4, and IFN-*α* decreasing and IFN-*γ* increasing after treatment (all *p* < 0.05).

**Conclusion:**

QCF treatment was associated with significant clinical improvement accompanied by oral microbiota remodeling and modulation of inflammatory cytokines. These findings support a potential microbiome-immune axis in PPP and warrant further controlled studies.

**Clinical trial registration:**

http://www.itmctr.org, ITMCTR2025001530.

## 1 Introduction

Palmoplantar pustulosis (PPP) is a chronic, recurrent inflammatory dermatosis confined to the palms and soles, characterized by clusters of sterile pustules, vesicles, erythematous plaques, lichenification, and abnormal desquamation (1). The prevalence of PPP varies by region, ranging from approximately 0.05% in Western populations to 0.12% in Japan. This disease predominantly affects adults, particularly women (accounting for 60–80% of cases), with the highest incidence observed in the 40–69 age group (mean age of onset 48–55 years) (2, 3). PPP imposes a substantial burden on patients’ quality of life, contributing to pain, pruritus, impaired hand-foot function, and psychosocial distress (4). Although current treatment strategies, including topical corticosteroids, phototherapy, and biologics, may provide symptomatic relief, their effectiveness is limited by frequent relapses, incomplete responses, and potential adverse effects (5).

Increasing evidence indicates that PPP pathogenesis involves innate immune activation and autoinflammatory signaling, particularly dysregulation of the IL-36 axis and downstream cytokine networks (6). In recent years, increasing attention has been given to the role of microbiota in inflammatory skin diseases (7, 8). The oral cavity harbors one of the body’s most complex microbial ecosystems, capable of shaping systemic immune responses through microbial metabolites, inflammatory mediators, and mucosal immune signaling (9, 10). Oral dysbiosis has been associated with psoriasis, atopic dermatitis, and other inflammatory dermatoses (11), suggesting a potential oral-skin inflammatory axis. However, the role of the oral microbiota in PPP pathogenesis, especially its dynamic changes in response to treatment, remains largely unexplored.

Within the framework of Traditional Chinese Medicine (TCM), PPP is typically categorized under “damp-heat accumulation” and “toxin retention,” reflecting pathological processes attributed to the retention of pathogenic dampness, heat, and toxins (12, 13). Therapeutic principles such as “clearing damp-heat” and “detoxification” aim to reduce inflammatory burden and restore physiological homeostasis. Herbal formulations based on these principles often contain bioactive components with reported anti-inflammatory, immunomodulatory, antioxidant, and microbiota-regulating properties (14, 15). Emerging pharmacological research further suggests that specific herbal compounds can modulate host–microbiome interactions, attenuate pro-inflammatory cytokines, and enhance epithelial barrier function (16, 17), providing a plausible mechanistic basis for TCM-mediated improvement of both skin inflammation and microbial balance.

Despite growing recognition of microbiota–immune interactions in dermatoses, studies investigating the oral microbiome in PPP are scarce, and longitudinal data assessing microbial changes before and after therapeutic intervention are lacking. Furthermore, no published work has systematically examined how TCM strategies rooted in the “damp-heat clearing and detoxification” principle affect oral microbial composition in PPP patients.

Given the limitations of existing treatments and the need for complementary therapeutic approaches, elucidating the microbiome-related mechanisms of TCM interventions may offer important clinical and mechanistic insights. Therefore, the present study aimed to characterize oral microbiota alterations in PPP patients and to explore potential microbiota-immune pathways associated with clinical improvement following an 8-week TCM regimen. These findings may provide new perspectives on the microecological basis of PPP and support the integration of microbiome modulation into TCM-based therapeutic strategies.

## 2 Materials and methods

### 2.1 Study design and participants

Thirty patients diagnosed with PPP according to established clinical criteria (18) were recruited from China Academy of Chinese Medical Sciences Affiliated Guang’AnMen Hospital between October 2022 and October 2024. Inclusion criteria were: (1) age>18 years; (2) active PPP lesions on palms and/or soles; and (3) no systemic antibiotics, probiotics, or immunosuppressive agents in the past 4 weeks. Exclusion criteria included: (1) concurrent systemic skin diseases; (2) having a known history of allergic reactions to a particular component of the therapeutic drug; and (3) severe systemic comorbidities. All participants provided written informed consent. The study was approved by the Ethics Committee of China Academy of Chinese Medical Sciences Affiliated Guang’AnMen Hospital (Approval No.2022-206-KY-01).

### 2.2 Intervention

All participants received an 8-week standardized Traditional Chinese Medicine (TCM) prescription based on the “dampness-heat clearing and detoxification” principle. The formula Qingpao Chushi Jiedu Fang (QCF) consisted of Smilax glabra Roxb. (Rhizoma Smilacis glabrae, *Tu Fu Ling*), Rehmannia glutinosa Libosch. (Radix Rehmanniae, *Di Huang*), *Paeonia suffruticosa* Andr. (Cortex Moutan, *Dan Pi*), Cynanchum paniculatum (Bge.) Kitag. (Radix Cynanchi Paniculati, *Quan Shen*), *Phragmites communis* Trin. (Rhizoma Phragmitis, *Lu Gen*), Scutellaria barbata D. Don (Herba Scutellariae Barbatae, *Ban Zhi Lian*), Phellodendron chinense Schneid. (Cortex Phellodendri Chinensis, *Huang Bai*), Hedyotis diffusa Willd. (Herba Hedyotidis Diffusae, *Bai Hua She She Cao*), *Pinellia ternata* (Thunb.) Breit. (Rhizoma Pinelliae Praeparata, *Qing Ban Xia*), Coptis chinensis Franch. (Rhizoma Coptidis, *Huang Lian*), *Euonymus alatus* (Thunb.) Sieb. (Cortex Euonymi, *Gui Jian Yu*), *Lonicera japonica* Thunb. (Caulis Lonicerae, *Ren Dong Teng*), Scutellaria baicalensis Georgi (Radix Scutellariae, *Huang Qin*), and Taraxacum mongolicum Hand.-Mazz. (Herba Taraxaci, *Pu Gong Ying*). The decoction was administered orally twice daily (150 mL each time). No systemic or topical treatments were permitted during the study period except for basic emollients.

### 2.3 Clinical assessments

#### 2.3.1 Palmoplantar Pustulosis Area and Severity Index (PPPASI)

PPP severity was evaluated at baseline and at weeks 2, 4, 6, and 8 using the PPPASI scoring system, which quantifies pustules, erythema, and scaling across palms and soles. Each domain was scored from 0 to 4, combined with lesion area to generate a total PPPASI score (range: 0–72). Scoring was performed independently by two dermatologists using standardized clinical photographs; discrepancies were resolved by consensus.

#### 2.3.2 Dermatology Life Quality Index (DLQI)

Quality of life was assessed at baseline and weeks 4, 6, and 8 using the validated 10-item DLQI questionnaire (score range 0–30). Higher scores indicate greater impairment. The Chinese-validated version was used.

#### 2.3.3 VAS for pain and pruritus

Pain and itch intensity were evaluated at baseline and each follow-up visit using a 10-cm visual analogue scale (0 = no symptom; 10 = worst imaginable symptom). Patients marked their symptom intensity on the scale under standardized instructions.

### 2.4 Blood sample collection and cytokine analysis

Fasting peripheral venous blood (5 mL) was collected at baseline and after 8 weeks of treatment. Blood was centrifuged at 3000 rpm for 10 min at 4 °C to isolate serum, which was aliquoted and stored at −80 °C. Serum concentrations of IL-1β, IL-4, IFN-*α*, and IFN-*γ* were quantified using the Luminex MagPix® System (Luminex Corporation, Austin, TX, USA), a high-throughput multiplex bead-based immunoassay platform. Detection was performed based on fluorescence signal acquisition according to the manufacturer’s instructions. All samples were run in duplicate, and standard curves (R^2^ ≥ 0.99) were generated for quantitative analysis. Inter-assay and intra-assay coefficients of variation (CVs) below 10% were considered acceptable.

### 2.5 Sample collection

Saliva samples and tongue-coating samples were collected in the morning before tooth brushing or food intake, both at baseline and week 8. Participants abstained from eating, drinking, smoking, or chewing gum for at least 2 h prior to collection. Samples were collected into sterile tubes, placed immediately on ice, and stored at −80 °C until DNA extraction.

### 2.6 DNA extraction and sequencing

Total microbial DNA was extracted using the QIAamp DNA Mini Kit (Qiagen, Germany). DNA quantity and purity were measured using a NanoDrop 2000 spectrophotometer, and integrity was verified by 1% agarose gel electrophoresis. The V3-V4 region of the 16S rRNA gene was amplified using primer 341F (5′-CCTACGGGNGGCWGCAG-3′) and 806R (5′-GGACTACHVGGGTWTCTAAT-3′). PCR products were purified with AMPure XP beads, quantified using a Qubit 4.0 fluorometer, pooled in equimolar concentrations, and sequenced in 1 × 300 bp mode on the Illumina MiSeq platform. Negative controls were included to monitor contamination.

### 2.7 Bioinformatics analysis

Raw reads were quality-filtered using FastQC and trimmed using Trimmomatic. Host DNA was removed using Kneaddata. Taxonomic profiling was performed using MetaPhlAn2 (17). *α*-diversity (Shannon index, observed ASVs, Faith’s phylogenetic diversity) and *β*-diversity (Bray–Curtis, weighted/unweighted UniFrac) were calculated in QIIME2. PCoA was used for visualization. Differentially abundant taxa were identified using LEfSe and DESeq2 (LDA > 2.0; *p* < 0.05).

### 2.8 Standardized clinical photography

Clinical images were captured using a Canon EOS 700D camera under controlled lighting (5,500 K LED), fixed distance (~30 cm), and neutral background. Camera angle, exposure, and settings were kept constant across all visits. Only uniform cropping was applied.

### 2.9 Statistical analysis

The normality of continuous variables was assessed using the Shapiro–Wilk test. For normally distributed data, results were expressed as mean ± standard deviation (Mean ± SD) and analyzed using paired *t*-tests. For non-normally distributed data, results were summarized as median and interquartile range [*M* (P25, P75)] and compared using Wilcoxon signed-rank tests. Continuous variables are additionally reported as mean ± SEM where appropriate for graphical presentation. Categorical variables were summarized as frequencies and percentages. Microbiota community-structure differences were evaluated using PERMANOVA based on Bray–Curtis and UniFrac distance matrices. A two-tailed *p* < 0.05 was considered statistically significant. Statistical analyses were performed in R (version 4.3.2) and Python (version 3.11).

## 3 Results

A total of 30 patients with PPP and 28 individuals serving as health controls were recruited, with complete 16S rRNA for saliva and tongue coating microbiome samples. The characteristics of the included subjects are shown in Supplementary Table S1.

### 3.1 Clinical improvements and symptom response to QCF treatment

**Figure 1:**
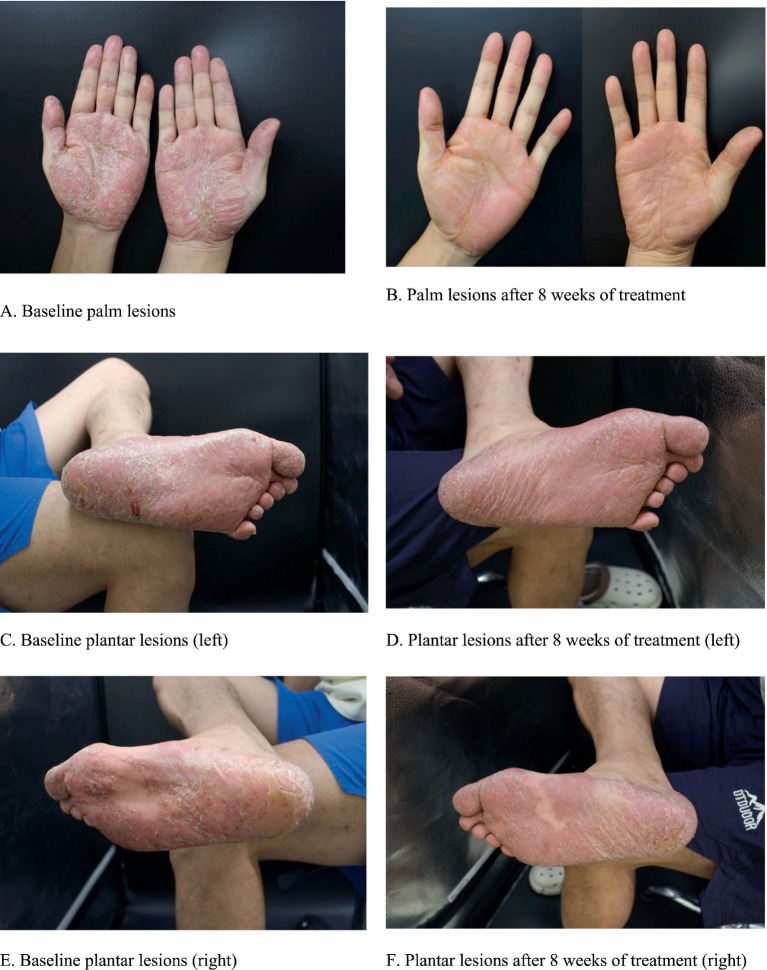
Clinical photographs of palmar and plantar lesions at baseline and after 8 weeks of treatment with *Qingpao Chushi Jiedu Fang*. **(A)** Clinical photographs of palmar lesions at baseline. **(B)** Clinical photographs of palmar lesions after 8 weeks of treatment with *Qingpao Chushi Jiedu Fang*. **(C)** Clinical photographs of right plantar lesions at baseline. **(D)** Clinical photographs of right plantar lesions after 8 weeks of treatment with *Qingpao Chushi Jiedu Fang*. **(E)** Clinical photographs of left plantar lesions at baseline. **(F)** Clinical photographs of left plantar lesions after 8 weeks of treatment with *Qingpao Chushi Jiedu Fang.*

Representative clinical images demonstrated notable resolution of pustules and erythema after 8 weeks of QCF treatment (Figure 1). Further improvement was observed at the 28-week follow-up (Supplementary Figure S1), and additional representative cases are shown in Supplementary Figure S2. No treatment-related adverse events were reported.

Consistent with the photographic evidence, PPPASI scores significantly decreased from baseline at weeks 2, 4, 6, and 8 (*p* < 0.001), with additional improvements at week 4 vs. week 2 and week 8 vs. weeks 4 and 6 (*p* < 0.001). DLQI scores also declined significantly at weeks 4, 6, and 8 (*p* < 0.05), with week-8 scores showing greater improvement than early time points (*p* < 0.05). Pain scores remained largely unchanged across the study period (*p* > 0.05), although week-6 scores were lower than week-2 (*p* < 0.05). Itch scores showed significant improvement only at week 8 compared with baseline (*p* < 0.05). (Table 1).

**Table 1:** Clinical severity scores and serum cytokine levels before and after Qingpao Chushi Jiedu Fang treatment.

Parameter	Time point	Value (Mean ± SD/*M*(P25, P75))
Clinical assessments		
PPPASI	Baseline	16.20 (11.33, 33.10)
	Week 2	11.40 (8.10, 27.60)*
	Week 4	11.05 (5.70, 19.60)*
	Week 6	8.60 (4.35, 18.05)*
	Week 8	7.25 (4.22, 11.38)*
DLQI	Baseline	7.00 (5.00, 13.75)
	Week 2	7.00 (3.75, 11.00)
	Week 4	6.00 (3.75, 11.00)*
	Week 6	5.00 (3.00, 9.25)*
	Week 8	5.50 (3.00, 7.75)*
Pain VAS	Baseline	4.00 (2.00, 5.25)
	Week 2	3.00 (0.00, 5.50)
	Week 4	2.50 (0.00, 7.00)
	Week 6	2.50 (0.75, 5.00)
	Week 8	2.00 (0.00, 5.00)
Itch VAS	Baseline	2.50 (0.00, 4.25)
	Week 2	3.00 (0.00, 5.00)
	Week 4	1.50 (0.00, 5.00)
	Week 6	0.50 (0.00, 5.00)
	Week 8	0.00 (0.00.3.25)*
Serum cytokine analysis		
IL-1β (pg/mL)	Baseline	3.12 (2.49, 3.85)
	Week 8	2.23 (2.13, 2.44)*
IL-4 (pg/mL)	Baseline	3.62 (3.36, 4.13)
	Week 8	3.43 (3.15, 3.71)*
IFN-α (pg/mL)	Baseline	4.17 ± 0.91
	Week 8	3.46 ± 0.59*
IFN-γ (pg/mL)	Baseline	2.29 (2.02, 2.91)
	Week 8	2.76 (2.53, 3.39)*

*p < 0.05 vs. week 0 (baseline); PPPASI, Palmoplantar Pustulosis Area and Severity Index; DLQI, Dermatology Life Quality Index; VAS, Visual Analogue Scale.

Serum cytokine analysis revealed significant reductions in IL-1β, IL-4, and IFN-*α* and a significant increase in IFN-*γ* after treatment (all *p* < 0.05), indicating modulation of systemic inflammatory activity (Table 1).

### 3.2 Microbial composition and relative abundance

Across both saliva and tongue-coating samples, the predominant genera at baseline were *Streptococcus*, *Prevotella*, *Veillonella*, *Neisseria*, and *Haemophilus*. Following QCF treatment, the relative abundances of *Prevotella* and *Veillonella* increased, while *Streptococcus* and *Neisseria* decreased. These consistent shifts were observed in both sample types (Supplementary Figure S3).

### 3.3 *Α*-Diversity

**Figure 2:**
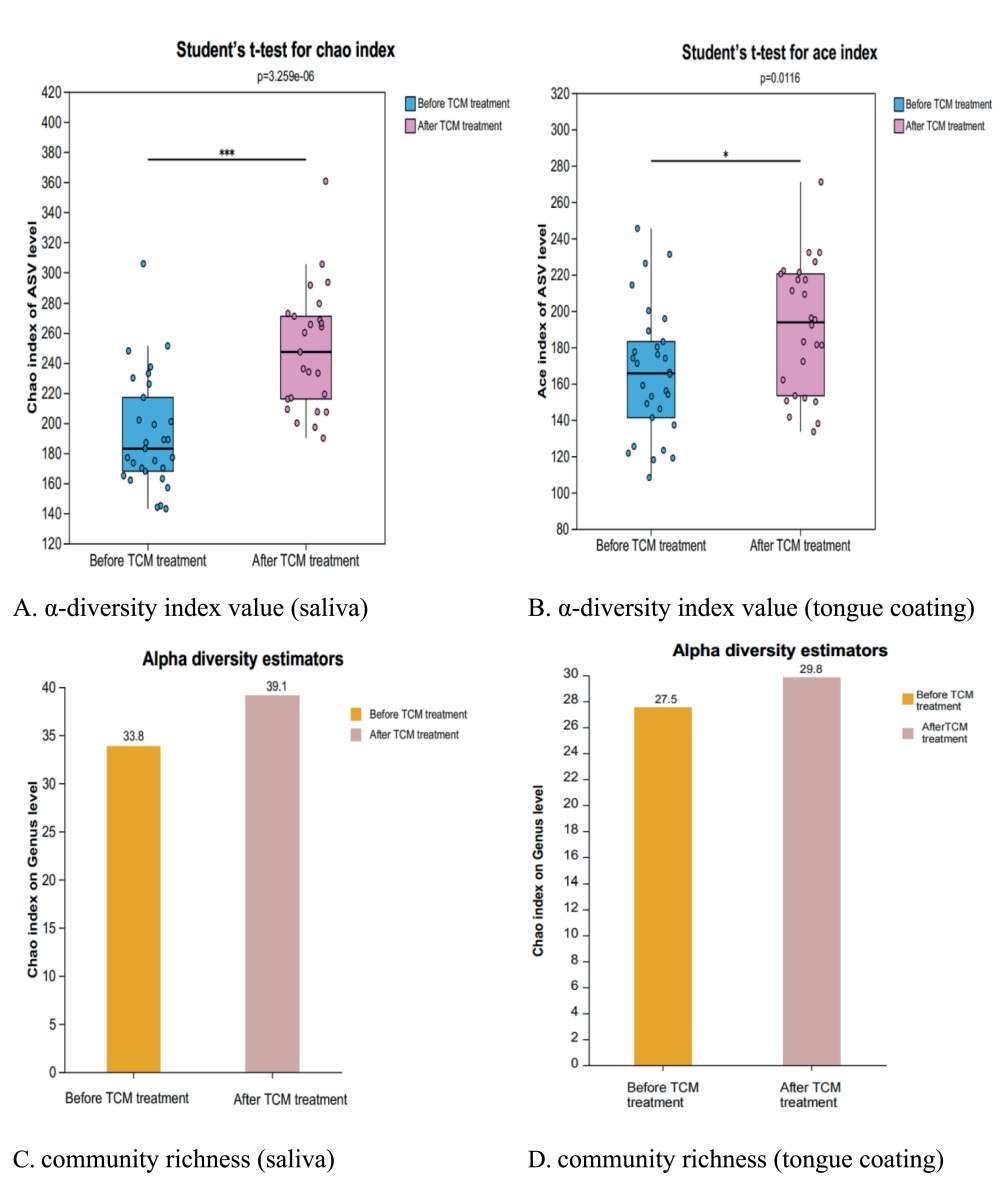
α-diversity comparisons of the oral microbiomes of the TCM treatment of PPP before and after. **(A)** α-diversity index difference in saliva sample. **(B)** α-diversity index difference in tongue coating sample. **(C)** Mean community richness in saliva sample. **(D)** Mean community richness in tongue coating sample; *p < 0.05; **p < 0.01; ***p < 0.001.

Significant changes in α-diversity were observed after treatment in saliva samples (*p* < 0.001) and tongue-coating samples (*p* < 0.05) (Figures 2A,B). In subgroup analyses stratified by smoking status, significant pre- to post-treatment differences were detected in salivary samples among smokers (Supplementary Figures S4A,B), whereas tongue-coating samples showed no significant subgroup differences (Supplementary Figures S4C,D).

Genus-level richness (Sobs index) showed similar patterns. Tongue-coating richness was comparable before (mean = 27.5) and after treatment (mean = 29.8), while salivary richness increased from 33.8 to 39.1 after treatment (Figures 2C,D).

### 3.4 *Β*-Diversity

**Figure 3:**
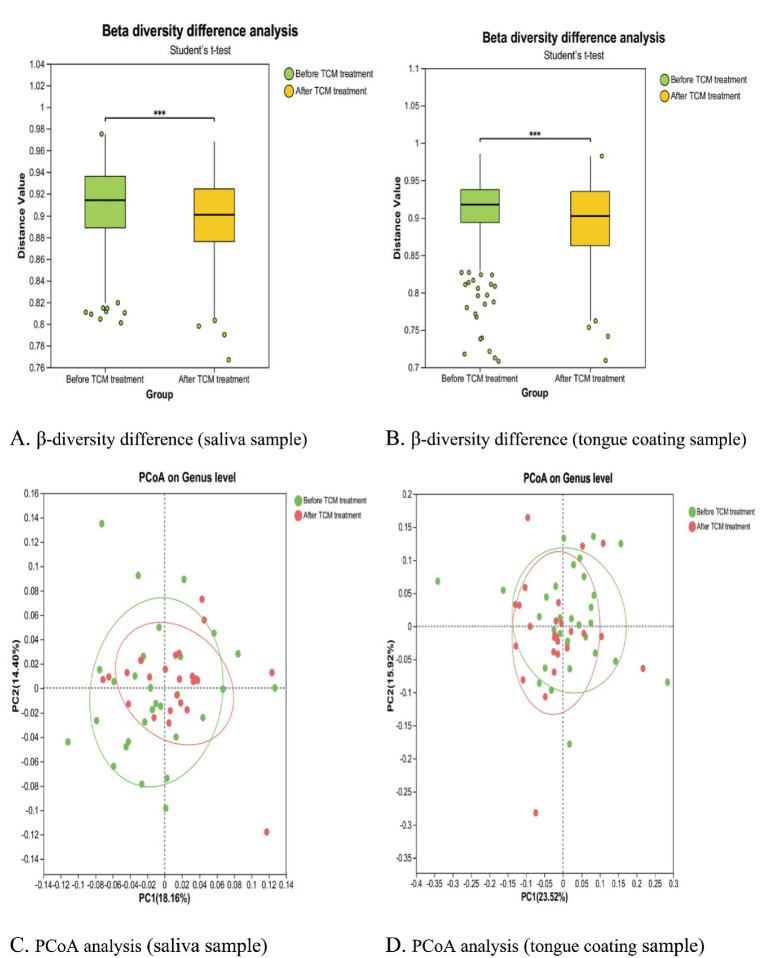
β-diversity comparisons of the oral microbiomes of the TCM treatment of PPP before and after. **(A)** β-diversity difference analysis in saliva sample. **(B)** β-diversity difference analysis in tongue coating sample. **(C)** β-diversity PCoA analysis in saliva sample. **(D)** β-diversity PCoA analysis in tongue coating sample; *p < 0.05; **p < 0.01; ***p < 0.001.

β-diversity analysis revealed significant differences between pre- and post-treatment samples for both saliva samples and tongue coating samples based on ASV-level metrics (*p* < 0.001; Figures 3A,B). In smoking-stratified analyses, significant shifts were identified among smokers in both sample types (*p* < 0.001; Supplementary Figures S5A,B), whereas no significant differences were observed among non-smokers (saliva samples: *p* = 0.241; tongue coating samples: *p* = 0.288; Supplementary Figures S5C,D).

PCoA plots showed distinct clustering patterns, with post-treatment samples displaying tighter clustering than baseline samples in both saliva samples and tongue-coating samples datasets (Figures 3C,D).

### 3.5 Community typing

In saliva samples, a baseline *Streptococcus*-dominant community shifted to a mixed *Streptococcus-Prevotella* pattern after treatment. In tongue-coating samples, the pre-treatment dominance of *Prevotella* and *Neisseria* transitioned to a *Prevotella*-enriched profile due to decreases in *Neisseria* (Supplementary Figure S6).

### 3.6 Differential taxa

**Figure 4:**
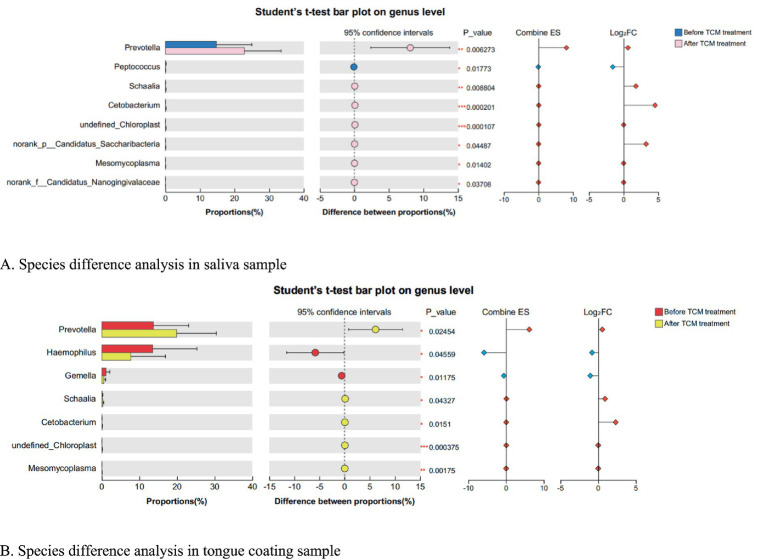
Species difference analysis of the oral microbiomes of the TCM treatment of PPP before and after. **(A)** Species difference analysis in saliva sample. **(B)** Species difference analysis in tongue coating sample; *p < 0.05; **p < 0.01; ***p < 0.001.

In saliva samples, *Prevotella*, *Schaalia*, *Cetobacterium*, and *Mesomycoplasma* were enriched after treatment, whereas *Peptococcus* was more abundant before treatment (*p* < 0.05). In tongue-coating samples, *Prevotella*, *Schaalia*, *Cetobacterium*, and *Mesomycoplasma* were also elevated post-treatment, while *Haemophilus* and *Gemella* were enriched at baseline (*p* < 0.05) (Figure 4).

## 4 Discussion

This study evaluated clinical outcomes, oral microbiota profiles, and inflammatory cytokine changes in PPP patients treated with the standardized TCM formula Qingpao Chushi Jiedu Fang. Consistent with observed improvements in pustules, erythema, scaling, and patient-reported symptoms, quantitative assessments demonstrated significant reductions in PPPASI, DLQI, and itch intensity over the 8-week treatment period. No treatment-related adverse events were reported, supporting the clinical tolerability of the regimen. These findings are noteworthy given the limited efficacy and recurrence risk associated with currently available therapeutic options for PPP, including topical corticosteroids, phototherapy, and systemic agents (19, 20).

Parallel to the clinical improvements, oral microbial diversity and community structure exhibited significant shifts across both saliva and tongue-coating samples. Increased *α*-diversity and convergent *β*-diversity patterns suggested a transition toward a more stable and less heterogeneous microbial community following treatment. Taxonomic analyses revealed consistent enrichment of genera such as *Prevotella*, *Schaalia*, *Cetobacterium*, and *Mesomycoplasma*, accompanied by reductions in *Streptococcus*, *Neisseria*, *Haemophilus*, and *Gemella*. These coordinated changes indicate broad reorganization of the oral microbiota rather than isolated taxonomic fluctuations, providing microbiological context for the observed clinical responses.

Serum cytokine measurements revealed decreases in IL-1β, IL-4, and IFN-*α* and an increase in IFN-*γ* after treatment, reflecting modulation of pro-inflammatory immune pathways (21–23). Although the study design does not permit causal inference, the temporal alignment between cytokine changes, microbial restructuring, and clinical improvement suggests an interconnected pattern of biological responses. Such concurrent shifts are consistent with an emerging model in which oral microbial communities contribute to systemic inflammatory tone and may influence cutaneous disease expression.

Differences identified between saliva samples and tongue-coating samples highlight the complementary nature of these oral niches, with saliva capturing dynamic microbial fluctuations and tongue coating representing a more stable biofilm-associated community. Addressing both environments strengthens the resolution of oral microbiome profiling and provides a more comprehensive view of treatment-associated microbial changes.

Smoking-stratified analyses further revealed that smokers exhibited more pronounced microbial restructuring than non-smokers. Given that smoking is known to alter oral microbial ecology and increase inflammatory signaling (24, 25), individuals with greater baseline dysbiosis may display a larger magnitude of microbiota shift during intervention. These findings emphasize the relevance of host lifestyle factors when evaluating microbiome-based responses and may inform personalized management strategies for PPP.

Several limitations should be acknowledged. The sample size was modest, which may restrict statistical power and limit generalizability. The absence of a control group precludes definitive attribution of microbial or cytokine changes to the intervention alone. Additionally, mechanistic pathways linking oral microbial alterations, cytokine modulation, and skin improvement were not directly assessed. Future research with larger cohorts, controlled designs, and integrative multi-omics, including metabolomics and cutaneous microbiome profiling, will be essential for elucidating causal pathways and clarifying the specific contributions of individual herbal constituents.

In summary, treatment with Qingpao Chushi Jiedu Fang was associated with improvements in clinical severity, oral microbial diversity and composition, and systemic cytokine profiles in patients with PPP. These findings suggest coordinated microbiome-immune responses during treatment and encourage further investigation into host–microbiota interactions as potential contributors to PPP pathophysiology.

## Data Availability

The data presented in the study are deposited in the SRA repository (RNS -Seq - SRA - NCBI), accession number PRJNA1395170.
