# Supplementary material for: Clinical and immunomicrobiome correlates of a standardized Qingpao Chushi Jiedu Fang regimen in palmoplantar pustulosis
Source: Front Med (Lausanne). 2026 Jul 3;13:1852035. doi: 10.3389/fmed.2026.1852035 (PMC13375502; doi:10.3389/fmed.2026.1852035)
Supplement: Supplementary file 1 [file Table_1.docx]

**Clinical and immunomicrobiome correlates of a standardized Qingpao Chushi Jiedu Fang regimen in palmoplantar pustulosis**

Junhui Wang, MD, PhD; Yannan Yang, MSc; Zehui Chen*, MD, PhD

**Supplementary Materials**

**Supplementary Table S1 Characteristics of the included subjects**

|  | Total | PPP patients | Health control |
| --- | --- | --- | --- |
| Age (mean±SD) | 37.2±12.8 | 46.3±11.5 | 27.6±4.6 |
| Gender |  |  |  |
| Male | 19 | 10 | 9 |
| Female | 39 | 20 | 19 |
| BMI (mean±SD) | 22.9±3.5 | 24.5±3.5 | 21.3±2.7 |
| Smoking |  |  |  |
| No | 43 | 18 | 25 |
| Yes | 15 | 12 | 3 |
| Alcohol |  |  |  |
| No | 46 | 23 | 23 |
| Yes | 12 | 7 | 5 |

*Abbreviation: PPP, palmoplantar pustulosis; SD, standard deviation*

| *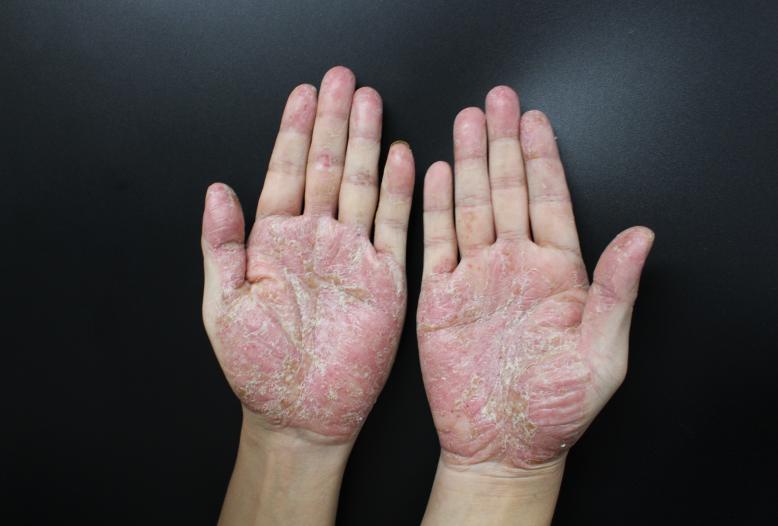*  A. Baseline palm lesions | *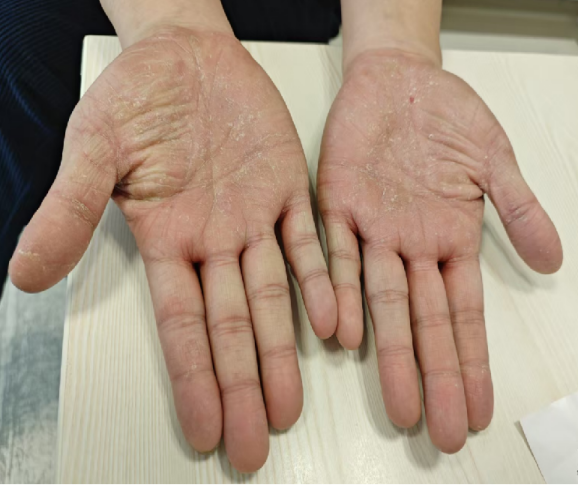*   1. Palm lesions after 28 weeks of treatment |
| --- | --- |
| *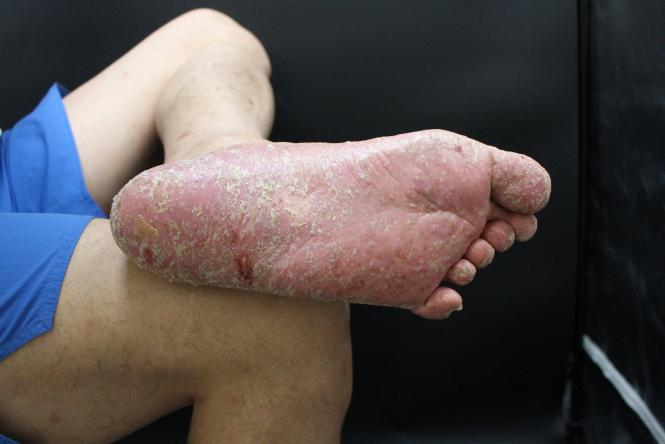*   1. Baseline plantar lesions (left) | *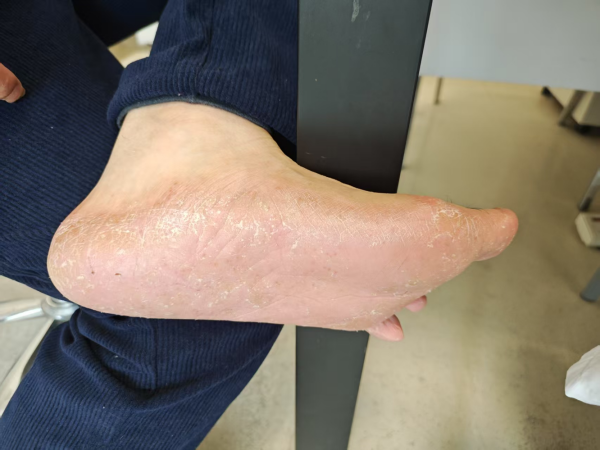*   1. Plantar lesions after 28 weeks of treatment (left) |
| *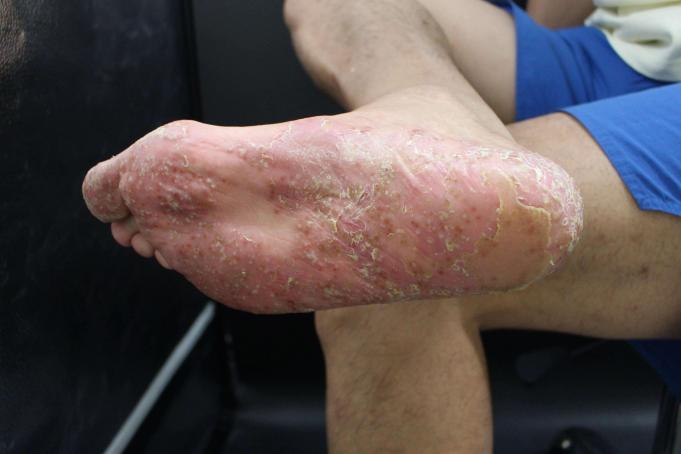*   1. Baseline plantar lesions (right) | *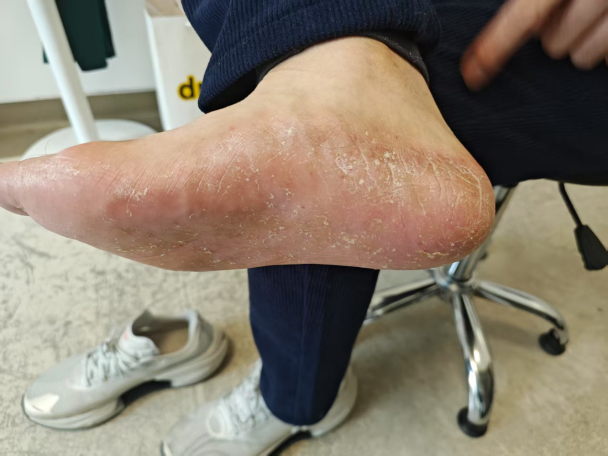*   1. Plantar lesions after 28 weeks of treatment (right) |

**Suppl. Figure S1 Clinical photographs of palmar and plantar lesions at baseline and after 28 weeks of treatment with *Qingpao Chushi Jiedu Fang***

1. A.Clinical photographs of palmar lesions at baseline; B. Clinical photographs of palmar lesions after 28 weeks of treatment with *Qingpao Chushi Jiedu Fang*; C. Clinical photographs of right plantar lesions at baseline; D. Clinical photographs of right plantar lesions after 28 weeks of treatment with *Qingpao Chushi Jiedu Fang*; E. Clinical photographs of left plantar lesions at baseline; F. Clinical photographs of left plantar lesions after 28 weeks of treatment with *Qingpao Chushi Jiedu Fang*

| *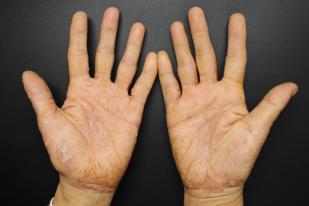*  A1. Baseline palm lesions | *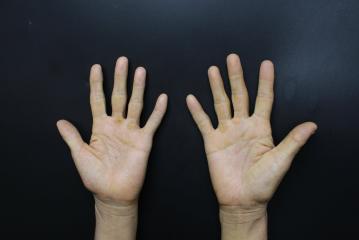*  B1. Palm lesions after 8 weeks of treatment | *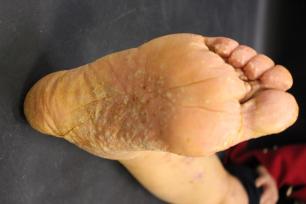*  C1. Baseline plantar lesions (right) | *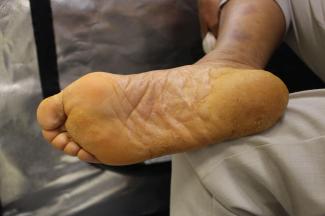*  D1. Plantar lesions after 8 weeks of treatment (right) |
| --- | --- | --- | --- |
| *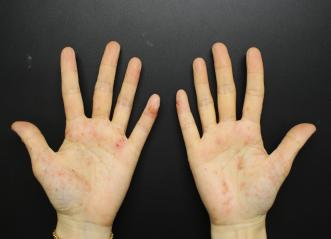*  A2. Baseline palm lesions | *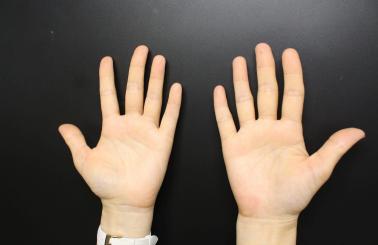*  B2. Palm lesions after 8 weeks of treatment | *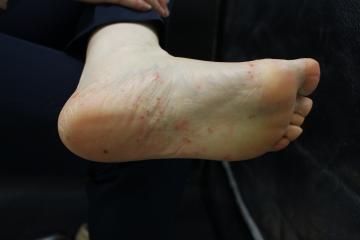*  C2. Baseline plantar lesions (left) | *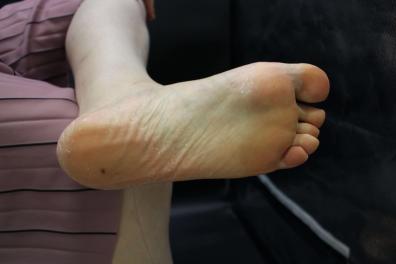*  D2. Plantar lesions after 8 weeks of treatment (left) |
| 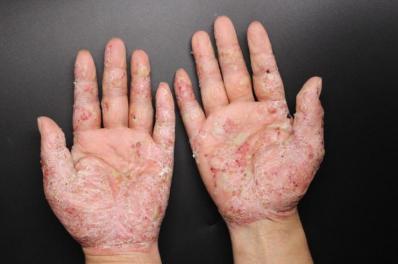  A3. Baseline palm lesions | 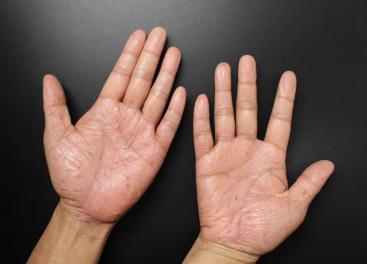  B3. Palm lesions after 8 weeks of treatment | 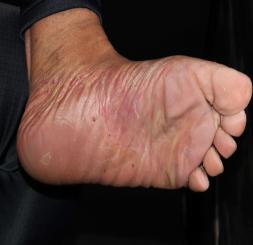C3. Baseline plantar lesions (left) | *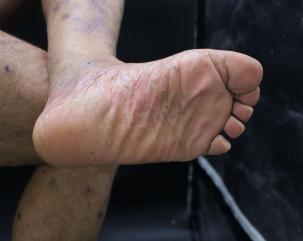*  D3. Plantar lesions after 8 weeks of treatment (left) |

**Suppl. Figure S2 Clinical photographs of palmar and plantar lesions at baseline and after 8 weeks of treatment with *Qingpao Chushi Jiedu Fang***

1. Clinical photographs of palmar lesions at baseline; B. Clinical photographs of palmar lesions after 8 weeks of treatment with *Qingpao Chushi Jiedu Fang*; C. Clinical photographs of plantar lesions at baseline; D. Clinical photographs of plantar lesions after 8 weeks of treatment with *Qingpao Chushi Jiedu Fang*; 1-3: Patients 1-3 with palmoplantar pustulosis

| 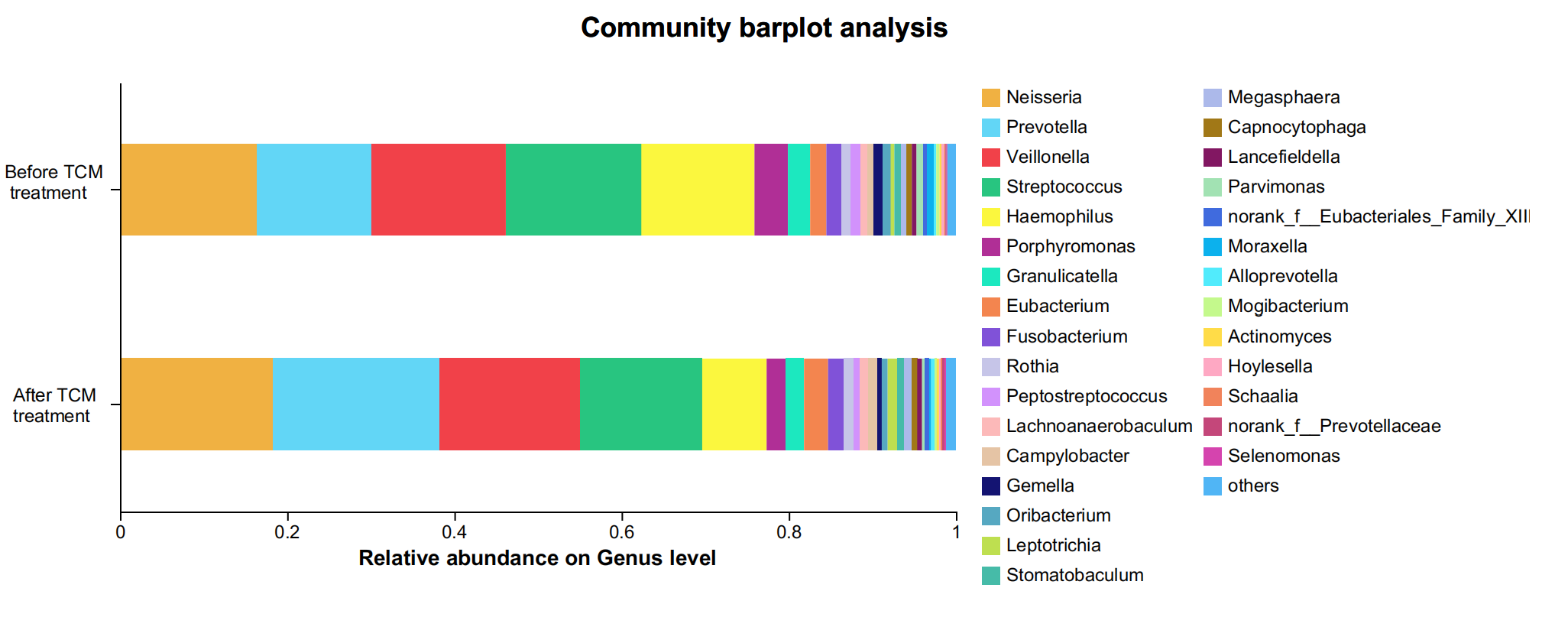  A.Tongue coating sample |
| --- |
| 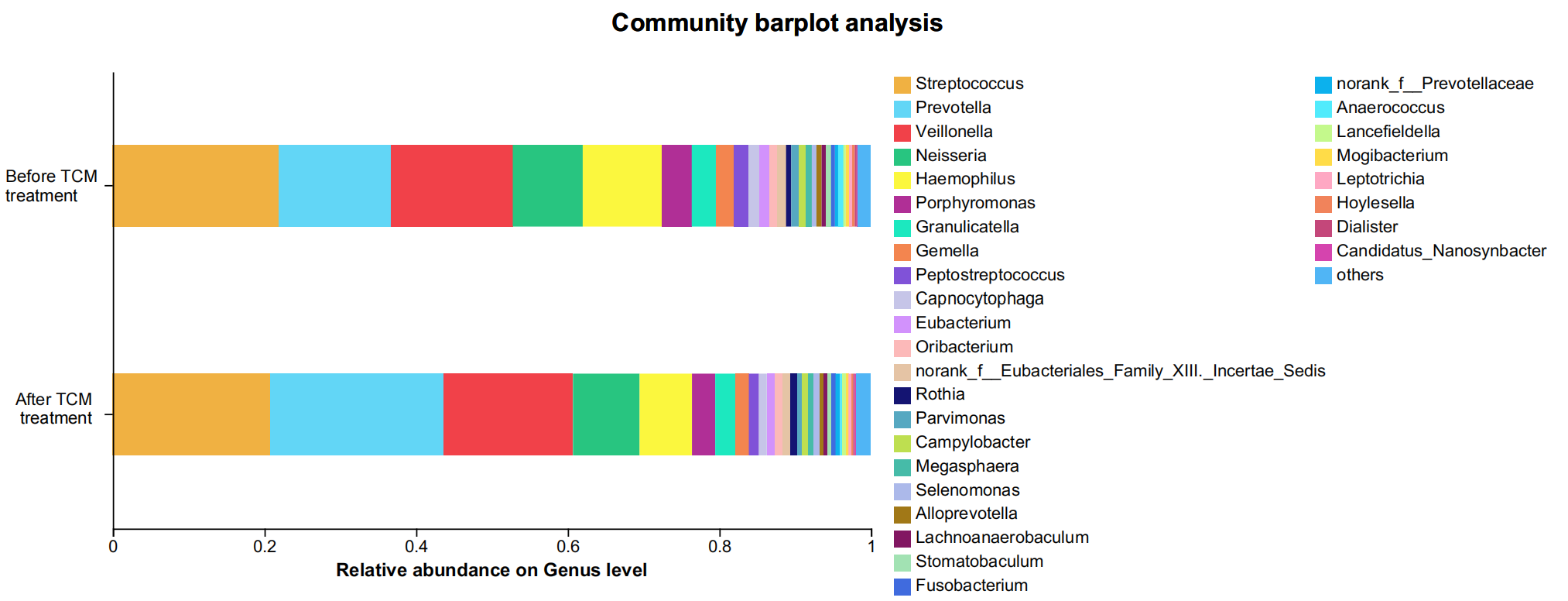   1. Saliva sample |

**Suppl. Figure S3 Microbial composition and relative abundance between TCM treatment of PPP before and after**

| 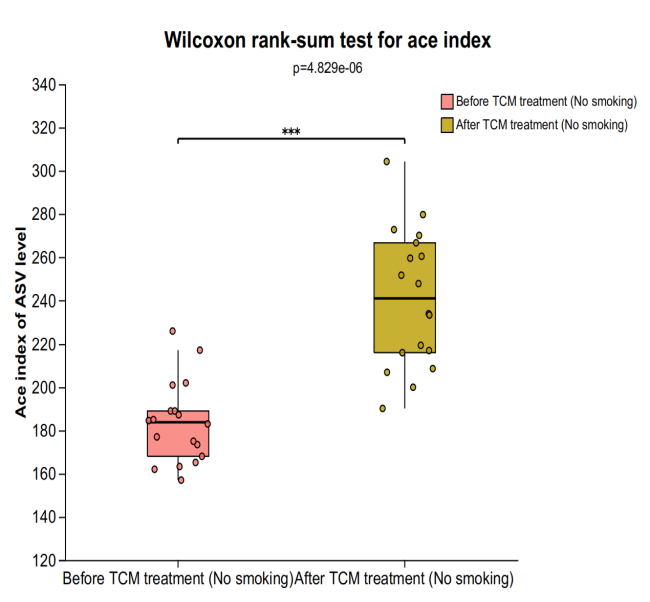   1. α-diversity index difference in saliva sample (no smoking) | 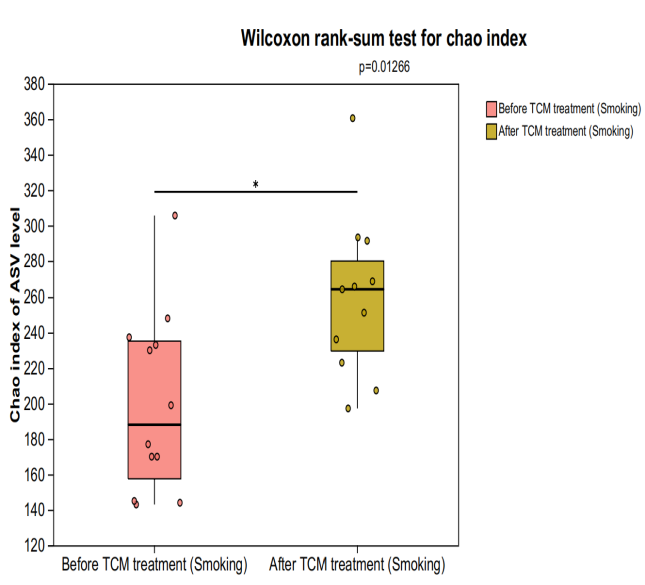   1. α-diversity index difference in saliva sample (smoking) |
| --- | --- |
| 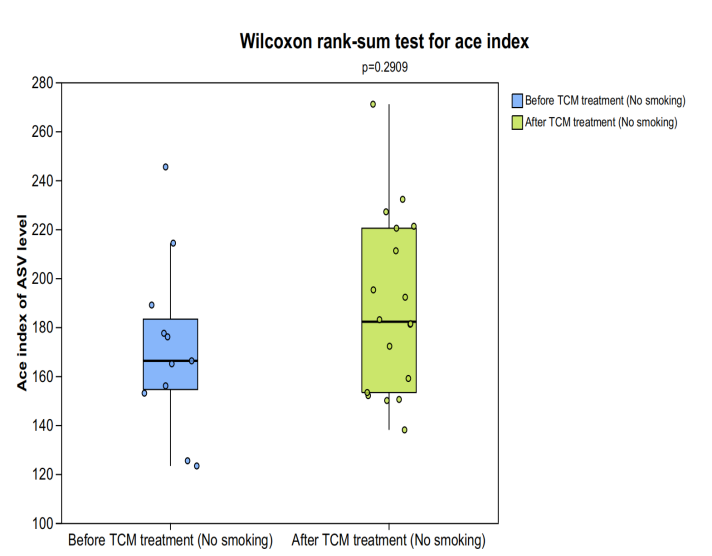   1. α-diversity index difference in tongue coating sample (no smoking) | 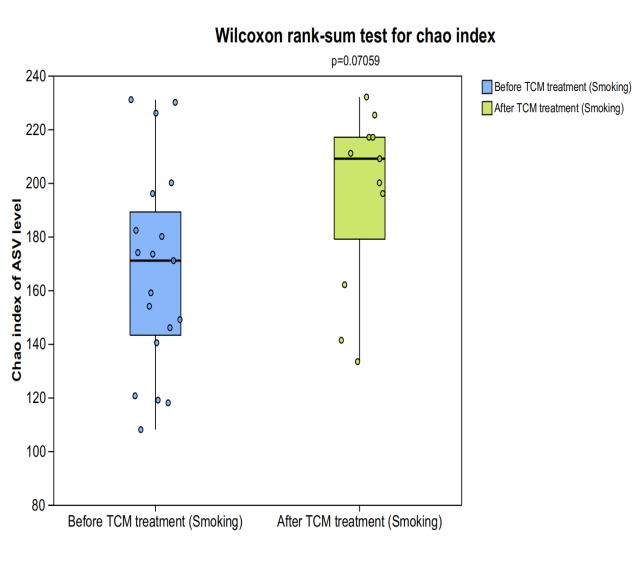   1. α-diversity index difference in tongue coating sample (smoking) |

**Suppl. Figure S4 Sub-group analysis of α-diversity index difference**

1. α-diversity index difference in saliva sample among no smoking status; B. α-diversity index difference in saliva sample among smoking status; C. α-diversity index difference in tongue coating among no smoking status; D. α-diversity index difference in tongue coating among smoking status; *p<0.05; **p<0.01; ***p<0.001

| 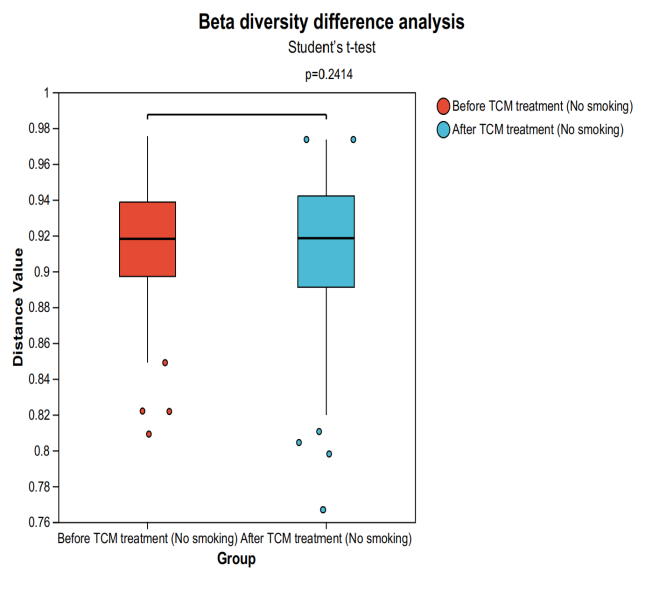   1. β-diversity difference analysis in saliva sample (no smoking) | 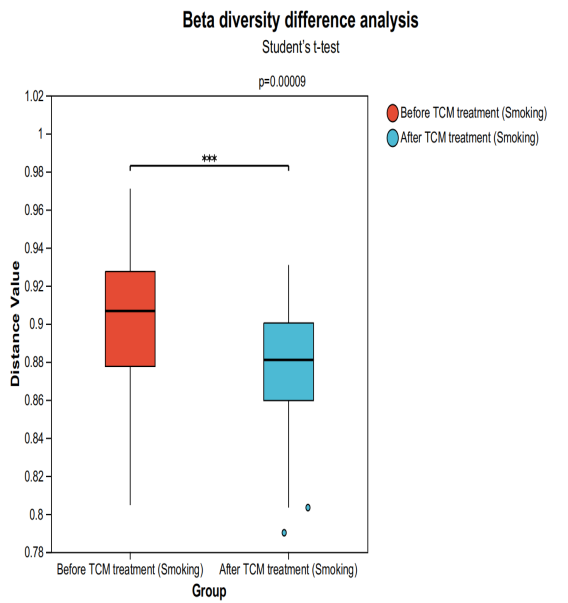   1. β-diversity difference analysis in tongue coating sample (no smoking) |
| --- | --- |
| 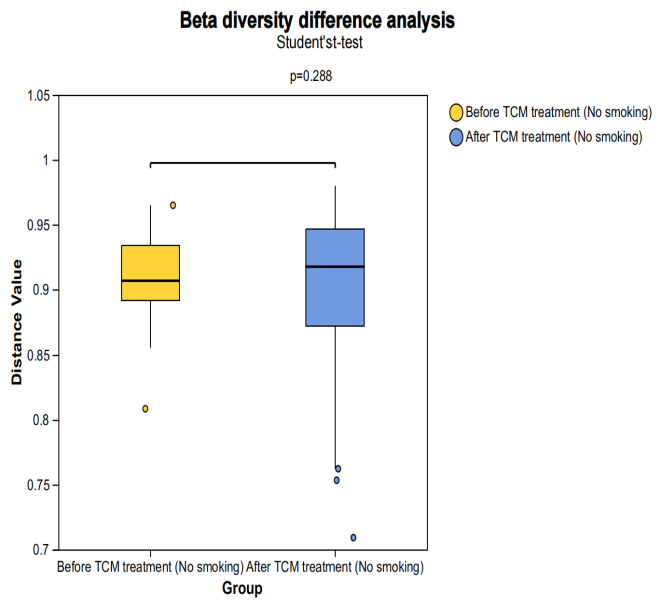   1. β-diversity difference analysis in saliva sample (smoking) | 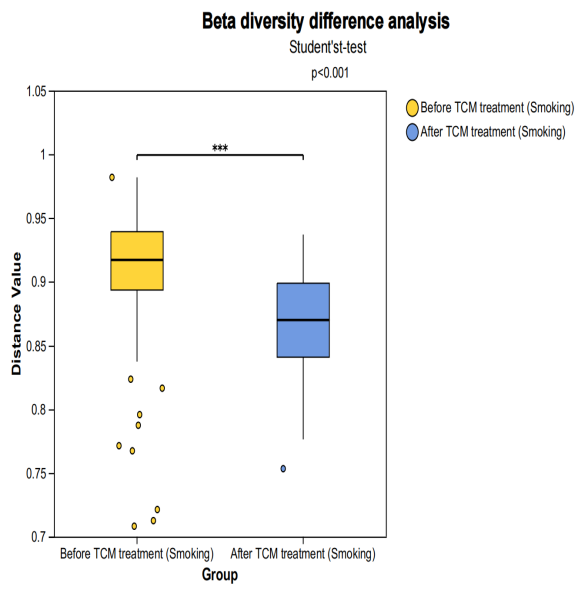   1. β-diversity difference analysis in tongue coating sample (smoking) |

**Suppl. Figure S5 Sub-group analysis of β-diversity difference analysis**

1. β-diversity difference analysis in saliva sample among no smoking status; B. β-diversity difference analysis in saliva sample among smoking status; C. β-diversity difference analysis in tongue coating among no smoking status; D. β-diversity difference analysis in tongue coating among smoking status; *p<0.05; **p<0.01; ***p<0.001

| 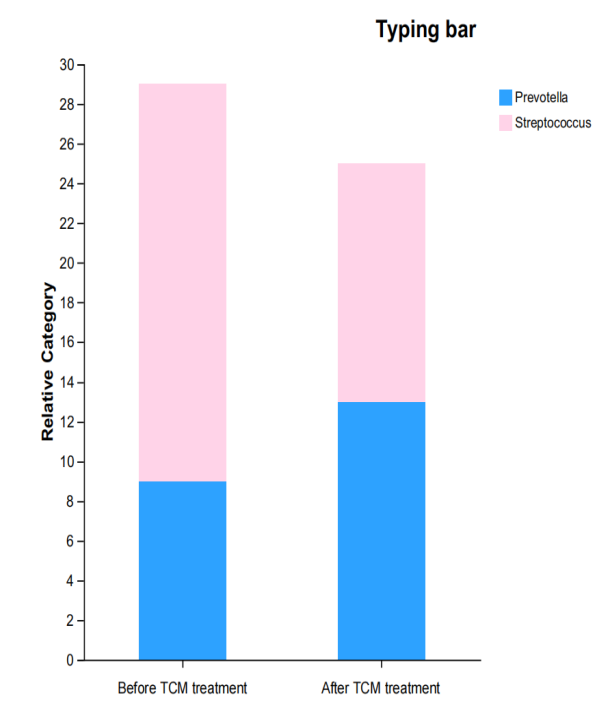   1. Saliva sample | 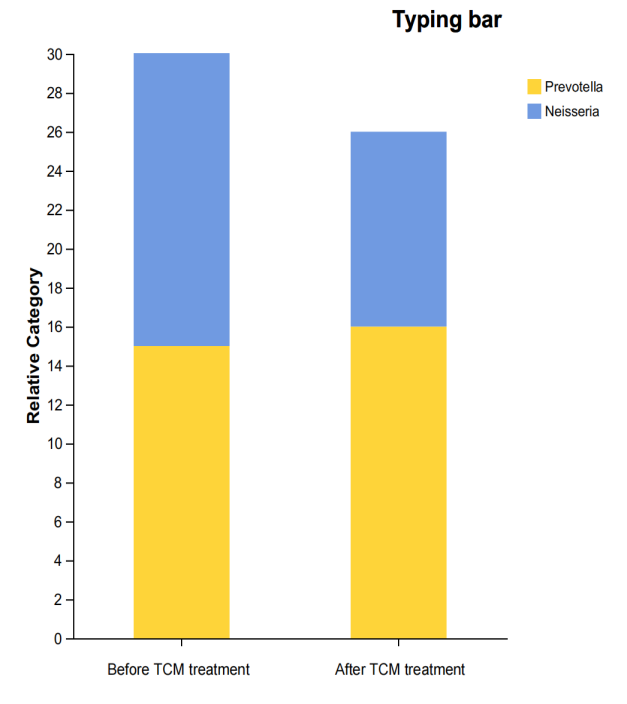   1. Tongue coating sample |
| --- | --- |

**Suppl. Figure S6 Community typing between TCM treatment of PPP before and after**
